# Molecular Taxonomy of Systemic Lupus Erythematosus Through Data-Driven Patient Stratification: Molecular Endotypes and Cluster-Tailored Drugs

**DOI:** 10.3389/fimmu.2022.860726

**Published:** 2022-05-09

**Authors:** Panagiotis Garantziotis, Dimitrios Nikolakis, Stavros Doumas, Eleni Frangou, George Sentis, Anastasia Filia, Antonis Fanouriakis, George Bertsias, Dimitrios T. Boumpas

**Affiliations:** ^1^ Laboratory of Autoimmunity and Inflammation, Biomedical Research Foundation of the Academy of Athens, Athens, Greece; ^2^ Rheumatology and Immunology, Hannover Medical School, Hannover, Germany; ^3^ Department of Gastroenterology, Academic Medical Center, Amsterdam Institute for Gastroenterology Endocrinology and Metabolism Amsterdam University Medical Center (UMC), University of Amsterdam, Amsterdam, Netherlands; ^4^ Department of Rheumatology and Clinical Immunology, Amsterdam Institute for Infection and Immunity, Amsterdam University Medical Center (UMC), University of Amsterdam, Amsterdam, Netherlands; ^5^ Amsterdam Rheumatology and Immunology Center (ARC), Academic Medical Center, Amsterdam, Netherlands; ^6^ Department of Experimental Immunology, Amsterdam Institute for Infection and Immunity, Amsterdam University Medical Center (UMC), University of Amsterdam, Amsterdam, Netherlands; ^7^ Department of Medicine, MedStar Georgetown University Hospital, Washington, DC, United States; ^8^ Department of Nephrology, Limassol General Hospital, Limassol, Cyprus; ^9^ Rheumatology Unit, First Department of Propaedeutic and Internal Medicine, National Kapodistrian University of Athens Medical School, Athens, Greece; ^10^ 4th Department of Internal Medicine, “Attikon” University Hospital, Athens, Greece; ^11^ Joint Rheumatology Program, Medical School, National and Kapodistrian University of Athens, Athens, Greece; ^12^ Department of Rheumatology, Clinical Immunology and Allergy, University of Crete School of Medicine, Heraklion, Greece; ^13^ Institute of Molecular Biology and Biotechnology, Foundation for Research and Technology – Hellas (FORTH), Heraklion, Greece

**Keywords:** molecular taxonomy, drug response prediction, systemic lupus erythematosus, drug repurposing, endotypes

## Abstract

**Objectives:**

Treatment of Systemic Lupus Erythematosus (SLE) is characterized by a largely empirical approach and relative paucity of novel compound development. We sought to stratify SLE patients based on their molecular phenotype and identify putative therapeutic compounds for each molecular fingerprint.

**Methods:**

By the use of whole blood RNA-seq data from 120 SLE patients, and in a data-driven, clinically unbiased manner, we established modules of commonly regulated genes (molecular endotypes) and re-stratified patients through hierarchical clustering. Disease activity and severity were assessed using SLEDAI-2K and Lupus Severity Index, respectively. Through an *in silico* drug prediction pipeline, we investigated drugs currently in use, tested in lupus clinical trials, and listed in the iLINCS prediction databases, for their ability to reverse the gene expression signatures in each molecular endotype. Drug repurposing analysis was also performed to identify perturbagens that counteract group-specific SLE signatures.

**Results:**

Molecular taxonomy identified five lupus endotypes, each characterized by a unique gene module enrichment pattern. Neutrophilic signature group consisted primarily of patients with active lupus nephritis, while the B-cell expression group included patients with constitutional features. Patients with moderate severity and serologic activity exhibited a signature enriched for metabolic processes. Mild disease was distributed in two groups, exhibiting enhanced basic cellular functions, myelopoiesis, and autophagy. Bortezomib was predicted to reverse disturbances in the “neutrophilic” cluster, azathioprine and ixazomib in the “B-cell” cluster, and fostamatinib in the “metabolic” patient subgroup.

**Conclusion:**

The clinical spectrum of SLE encompasses distinct molecular endotypes, each defined by unique pathophysiologic aberrancies potentially reversible by distinct compounds.

## Introduction

Systemic lupus erythematosus (SLE) has a unique set of attributes, which has established it as the prototype among systemic autoimmune diseases. With few notable exceptions, recent advances in the understanding of SLE pathogenesis have failed to translate into new therapies. High-throughput methods have enabled the discovery of novel drugs in a time- and cost-efficient manner. To this end, the Connectivity Map (CMap) project is the first powerful drug repurposing platform that embedded gene expression responses of 4 human cell lines treated with different doses of a large collection of FDA-approved compounds (1). Taking a step forward, the NIH-supported Library of Integrated Network-Based Cellular Signatures (LINCS) enriched the transcriptomic databases of the CMap project by integrating the gene expression profiles of more than 60 cell lines before and after exposure to more than 20,000 perturbagens (2). In this context, Toro-Dominguez et al. employed the successor of the CMap, Lincscloud, suggesting the therapeutic potential of phosphoinositol 3 kinase and mammalian target of rapamycin (mTOR) inhibitors in SLE (3).

We have previously used mRNA sequencing to define the transcriptomic signature of SLE patients. Our data showed that SLE is characterized by a “susceptibility signature” present in patients in clinical remission compared to healthy controls. Additionally, we identified an “activity signature” present in patients with active disease, which was mainly associated with genes that regulate immune cell metabolism, protein synthesis and proliferation. Lastly, we detected a “severity signature”, best illustrated in active nephritis, linked to granulocyte and plasmablast/plasma–cell pathways (4).

In the present study, we used the same RNA-sequencing dataset in order to stratify lupus patients according to underlying fundamental molecular aberrancies and predict personalized therapeutic options. Specifically, we established an *in silico* drug prediction pipeline to select the optimal treatments for each patient subgroup, among compounds that have already been tested against SLE in clinical trials. We also deployed a personalized drug repurposing pipeline to identify FDA-approved drugs or patented compounds for different indications, that could be applied as potential therapeutic agents for each group of SLE patients. We provide a comprehensive, in-depth analysis of the human SLE transcriptome to guide precision care and new therapeutic compound development.

## Materials and Methods

### Patients

Whole blood transcriptional profiles of 120 patients with SLE and 58 healthy individuals (4) were analyzed. Disease activity at the time of blood sampling was assessed by the modified Systemic Lupus Erythematosus Disease Activity Index 2000 (SLEDAI-2K), after exclusion of the serologic features (anti-dsDNA and complement levels) (clinical SLEDAI) (5). Remission was defined as a clinical SLEDAI-2K = 0 and daily prednisolone dose of ≤5 mg (6, 7). Active disease was defined as a clinical SLEDAI-2K ≥4. Irreversible organ damage was assessed using the SLICC damage index (SDI) (8). Lupus Severity Index was calculated for each patient (9).

### Co-Expression Network Analysis

We employed CoCena² (construction of co-expression network analysis-automated, https://github.com/UlasThomas/CoCena2), using the 10,000 most variable genes as input, to determine modules of co-expressed transcripts. Next, agglomerative hierarchical clustering of patients, based on their group fold changes (GFC) for each cluster of co-expressed genes, defined the disease molecular endotypes. Functional enrichment analysis was performed using clusterProfilerR package (10).

### Drug Prediction Analysis

DEseq2 was used to identify differentially expressed genes (DEGs) specific for each patient’s endotype (11). We obtained gene expression signatures of drugs that are incorporated in the treatment recommendations for SLE (12), or have failed to reach SLE clinical trials endpoints and are included in the following iLINCS sublibraries: i) iLINCS chemical perturbagens (LINCSCP); ii) iLINCS targeted proteomics signatures (LINCSTP); iii) Disease-related signatures (GDS); iv) Connectivity Map signatures (CMAP); v) DrugMatrix signatures (DM); vi) Transcriptional signatures from EBI Expression Atlas (EBI); vii) Cancer therapeutics response signatures (CTRS); and viii) Pharmacogenomics transcriptional signatures (PG). These were downloaded using the iLINCS API (https://github.com/ucbd2k/ilincsAPI/blob/master/usingIlincsApis.Rmd). Statistically significant DEGs from each drug signature were ordered by decreasing fold change magnitude. The top 300 DEGs were selected and upregulated/downregulated genes were identified. Gene set enrichment analysis (GSEA) was performed using fgsea R package (13). To determine the optimal number of drug clusters for k-means clustering, the elbow method was applied.

### Drug Repurposing Analysis

Drug repurposing results were prioritized using the bioinformatic tool CoDReS (Computational Drug repositioning score) (14), which enables the exploration of compound drugability, based on an algorithm that combines functional and structural scores. The functional score quantifies the pharmacodynamic potential of a compound by assessing its association to SLE hallmarks. This potential includes the binding affinity to SLE molecular targets (enzyme, receptor, transcription factor, etc.), as well as the overlap of its genomic targets with genes implicated in the pathogenesis of the disease. The structural score pertains to the pharmacokinetic properties of compounds and contains information related to the hydrophilic-lipophilic balance, solubility, permeability, as well as oral bioavailability of a drug candidate, based on the “Lipinski rules of 5” (15) and “Veber’s rule” (16).

## Results

### Co-Expression Analysis Stratifies SLE Patients Into Distinct Endotypes in an Unbiased Data-Driven Manner

**Figure 1:**
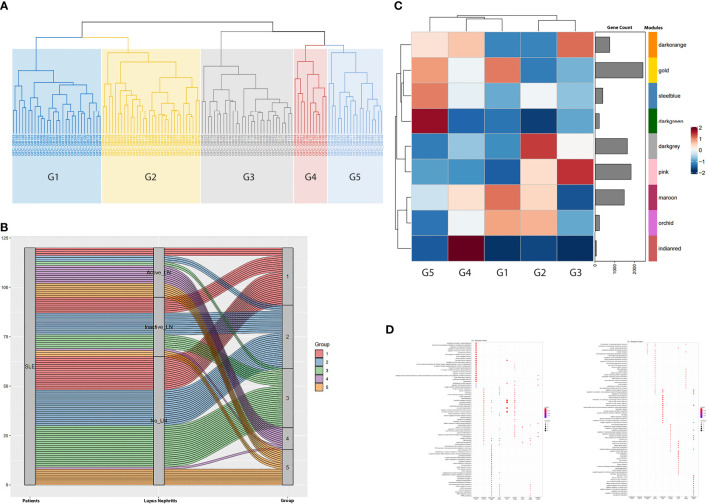
**(A)** Hierarchical clustering of the samples based on the magnitude of the expression of each gene module (identified in **Figure S1**) defined five groups of patients (G1 to G5). Briefly, the x-axis demonstrates the patients analyzed in our study. GFC denotes the Group Fold Changes (defined in **Figure S1**); GB denotes the sample; the number after the GB acronym denotes each patient database ID. **(B)** Alluvium plot illustrating the distribution of the SLE patients into the patient groups G1-G5 generated after the hierarchical clustering of the samples according to each module’s group fold changes. Briefly, the 120 SLE patients included in our study are displayed in the left vertical box (Patients). Each horizontal block corresponds to a patient. The distribution of the patients according to the presence and the activity of Lupus Nephritis (LN) was demonstrated in the middle vertical box. The distribution of the patients into the five CoCena2 analysis defined patient groups was shown in the right vertical box. **(C)** Heatmap showing the mean of the GFCs of the CoCena2 analysis derived gene modules in each one of the previously defined patient groups. Group specific GFCs demonstrated similar and counteracting gene expression patterns among patient groups. Briefly, increased expression of the indian-red module characterized G4. Enrichment of the dark-green module defined G5. Heightened expression of the dark-grey module distinguished G2. Lastly, enrichment of the pink and dark-orange modules was indicative of G3. **(D)** Dot plot displaying the functional enrichment analysis of the CoCena2-derived modules. Gene modules are shown on the basis of the graph. Enriched gene ontologies and pathways are shown on left side of the graph. Briefly, the indian-red module included genes that were mainly enriched in neutrophil activation and degranulation. Functional enrichment analysis of the dark-green module revealed disturbances related to plasmablast-mediated responses. Dark-grey module predominantly consisted of genes related to autophagy. Genes of the pink module were enriched in mRNA splicing, whereas gene ontologies related to mitochondrial function were overrepresented among the genes included in the dark-orange module.

Applying the CoCena² pipeline, we identified nine modules of co-expressed transcripts illustrated with different colors in **Figure S1**. Hierarchical clustering of samples according to each module’s group fold changes (GFC) reassigned patients into five groups (G1 to G5) (**Figures 1A, B**). To define disease-driving molecular mechanisms, we investigated the CoCena²-derived modules enrichment in each patient group (**Figures 1C, D**). Interestingly, groups displayed distinct enrichment patterns, each exhibiting unique major module predominance. Platelet activation and hemostasis were identified as two group 1 specific signals (G1, “Hemostasis” group), overrepresented in the *orchid module*. Detailed functional enrichment analysis of the *dark-grey module* revealed that autophagy-associated signatures were prominently enriched in patient group 2 (G2, “Autophagy” group). Macroautophagy disturbances in G2 are accompanied by deregulation of pathways involved in neutrophil activation and toll-like receptor (TLR) cascade. Combined enrichment of the *pink module*, linked to aberrancies of mRNA splicing and mRNA surveillance mechanisms, and the *dark-orange module*, implicated among others in mitochondrial dysfunction, efficiently distinguished group 3 (G3, “Metabolism” group). Heightened expression of the *indian-red module*, which predominantly consists of genes implicated in neutrophil activation and degranulation, defines group 4 (G4, “Neutrophil” group). Enrichment of the *dark-green module*, which comprises genes (such as CD38, BLNK, IGHA1, TNFRSF17, CD22, CD79A, MS4A1, IGHD) linked to B-cell and plasmablast-mediated responses, was indicative of group 5 (G5, “B cell” group). Interestingly, G5 displays a concurrent increased expression of the steel-blue module, which is associated with type I interferon (IFN) signaling.

### Molecular Clusters Are Associated With Distinct Clinical Traits

**Figure 2:**
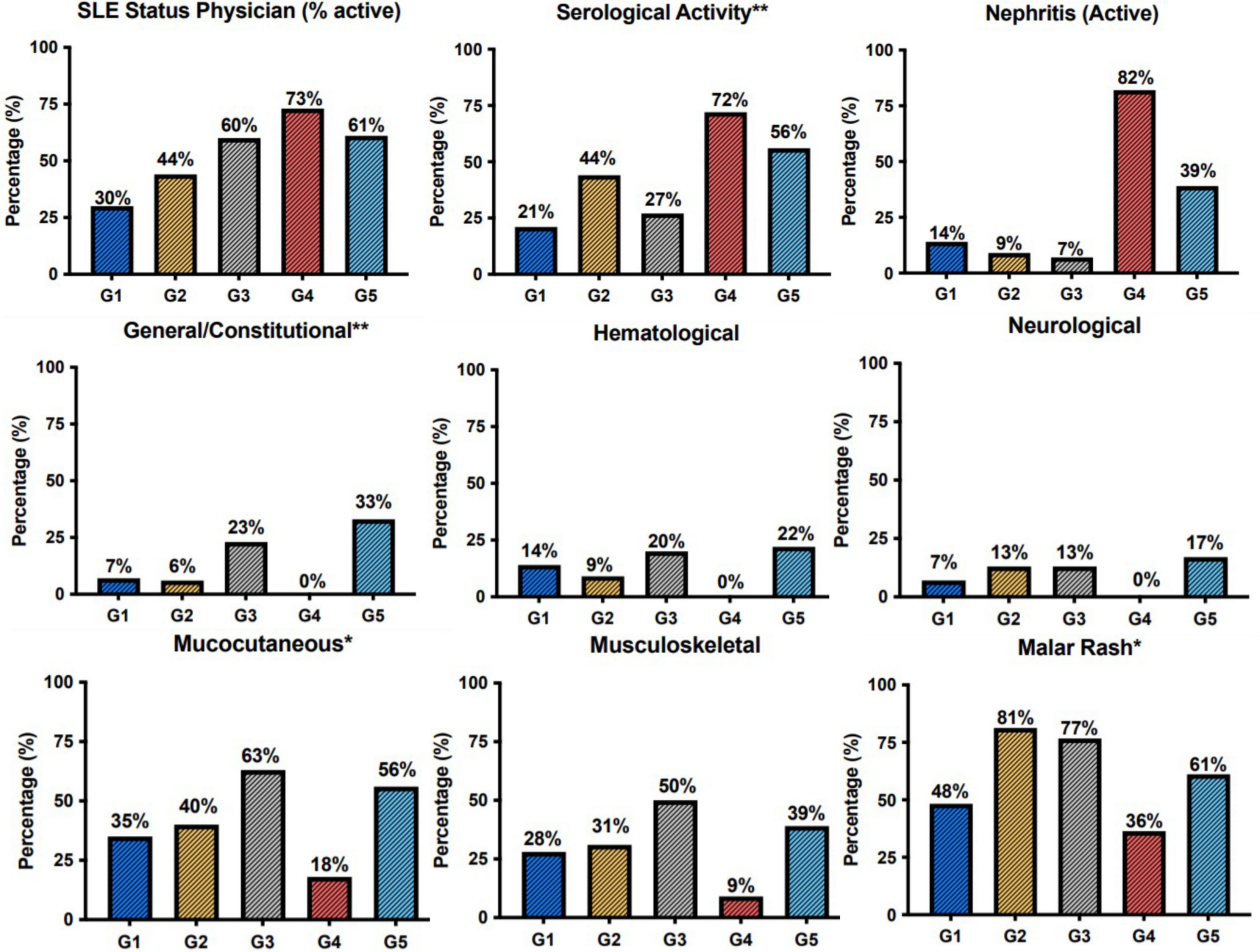
Barplots demonstrating the prevalence of clinical features, Physician Global Assessment (SLE.status.(Physician)) and serological activity across patient groups. The G4 was defined by the high prevalence of active lupus nephritis. Constitutional symptoms occurred most frequently in the G5. Mucocutaneous and musculoskeletal manifestations were more prevalent among patients of the G3. *p < 0.05; **p < 0.01 in Kruskal-Wallis test, Chi-squared test.

To evaluate the clinical implications of molecular endotype characterization, we next assessed each group’s clinical features, including demographics, clinical manifestations, serologic features, and administered treatments. The “Neutrophil” group (G4, n= 11, 9.1% of the total cohort) almost uniformly encompassed patients with active lupus nephritis (n=9/11) (**Figure 2**). Patients of this cluster also exhibited high serologic and clinical activity; the majority were treated with cyclophosphamide at the time of blood sampling (**Figures 2**, **S2**, **S3**). The “B-cell” group (G5, n=18, 15% of the total cohort) was characterized by high prevalence of constitutional symptoms. Although statistical significance was not reached, a tendency to a higher frequency of hematological and neurological manifestations was apparent in this cluster. Mucocutaneous and musculoskeletal manifestations were most common in the “Metabolism” group (G3, n=30, 25% of the total cohort), occurring in 63% and 50% of patients, respectively, while a history of neuropsychiatric SLE (NPSLE) was reported in 27%. Interestingly, the clinically heterogenous “Hemostasis” group (G1, 24.2% of the total cohort) was characterized by high frequency of male patients, while Disease Modifying Anti-Rheumatic Drugs (DMARDs) were the most commonly used therapy. Finally, the “Autophagy” group (G2, n=32, 26.7% of the total cohort) consisted of patients with mild to moderate SLE. Accordingly, photosensitivity and malar rash were found in 59,3% and 81,2% of the patients of G2, respectively.

### Molecular Endotypes Can Be Used to Predict Group-Specific Effective Compounds Towards Personalized Therapeutic Decisions

To explore personalized therapeutic solutions, we identified compounds tailored to each group’s molecular fingerprint. This was achieved through leveraging our CoCena² based co-expression analysis, to establish an *in silico*, signature-based, drug prediction pipeline. As group-specific signatures, we employed the DEGs resulting from the comparison of each SLE endotype with a pool of 58 healthy controls.

To this end, we initially collected the transcriptional profiles corresponding to cellular responses against drugs that are either currently used in clinical practice, are or have failed in SLE clinical trials and are listed in the iLINCS prediction databases (**Table S1**). Our query returned 3,900 drug signatures (**Table S2**). Using SLE group-specific transcriptional profiles as input, we performed GSEA against the datasets of the top upregulated and top downregulated DEGs for each drug signature and normalized enrichment scores (NES) were defined. Next, we calculated the difference (ΔNES) of the NES from the downregulated gene set and the NES from the upregulated gene set for each drug signature per SLE cluster (**Table S3**). Accordingly, a positive ΔNES indicated compounds that were predicted to reverse the group-specific transcriptomic aberrancies. To determine endotype-specific drug candidates, we applied k-means clustering, in order to group drug signatures according to ΔNES (**Figures S4**, **S5**). Drug signatures with the highest ΔNES within each drug cluster induce cellular transcriptional alterations which most efficiently counteract group-specific SLE signatures.

**Figure 3:**
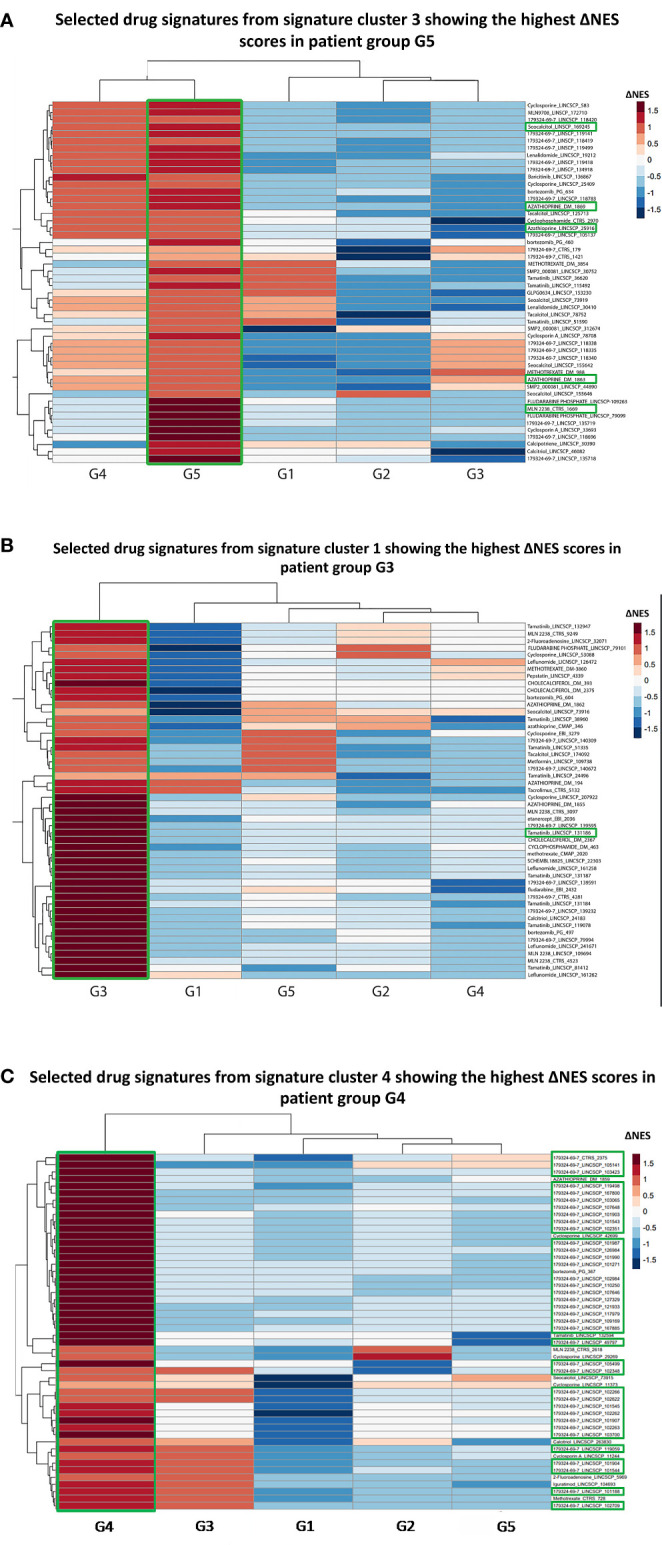
**(A)** Heatmap of the selected top 50 drug signatures from signature cluster 3 (**Figure S4**) showing the highest ΔNES score in the G5 patient group. Signatures of the azathioprin and the ixazomib showed the highest ΔNES scores in the G5 patient group. MLN2238: Ixazomib. Labeling was carried out based on the following strategy: “drug name”_”database”. **(B)** Heatmap of the selected top 50 drug signatures from signature cluster 1 with the highest ΔNES score in the G3 patient group. Signatures of SYK kinase inhibitor tamatinib showed the highest ΔNES scores in the G3 patient group. **(C)** Heatmap of the selected top 50 drug signatures from signature cluster 4 with the highest ΔNES score in the G4 patient group. 76% of the top 50 drug signatures for G4 patient group belonged to the proteasome inhibitor bortezomib. 179324-69-7: Bortezomib.

In G5, the top signatures were linked to azathioprine (ΔNES=2.76) and ixazomib (ΔNES=2.67) (**Figure 3A**), whereas in G2 to the proteasome inhibitor bortezomib (ΔNES=2.84). Signatures related to the SYK kinase inhibitor tamatinib (ΔNES=2.81) were top ranked in G3 subgroup (**Figure 3B**). In G4 group, signatures related to bortezomib occurred in high frequency (76%) among the top 50 signatures, starting with a ΔNES score 2.54 and, together with the calcineurin inhibitor cyclosporine (ΔNES score 2.49), might represent alternative G4-specific therapeutic options (**Figure 3C**). Finally, in both groups 4 and 5, signatures related to vitamin D derivatives (such as seacalcitol) prevailed, with a ΔNES score 3.04 and 2.97, respectively.

Since the majority of the G4 patients were treated with cyclophosphamide at sampling, an agent that could drastically alter the whole blood transcriptional landscape, we divided G4 into two subgroups; one treated with cyclophosphamide (G4A, n=6/11) and a “cyclophosphamide-free” subgroup (G4B, n=5/11) and we applied the drug prediction pipeline. In accordance with our initial findings, bortezomib was overrepresented among the top 10 signatures in both subgroups (**Tables S4**, **S5**).

### Drug Repurposing Tailored to SLE Molecular Aberrancies

**Figure 4:**
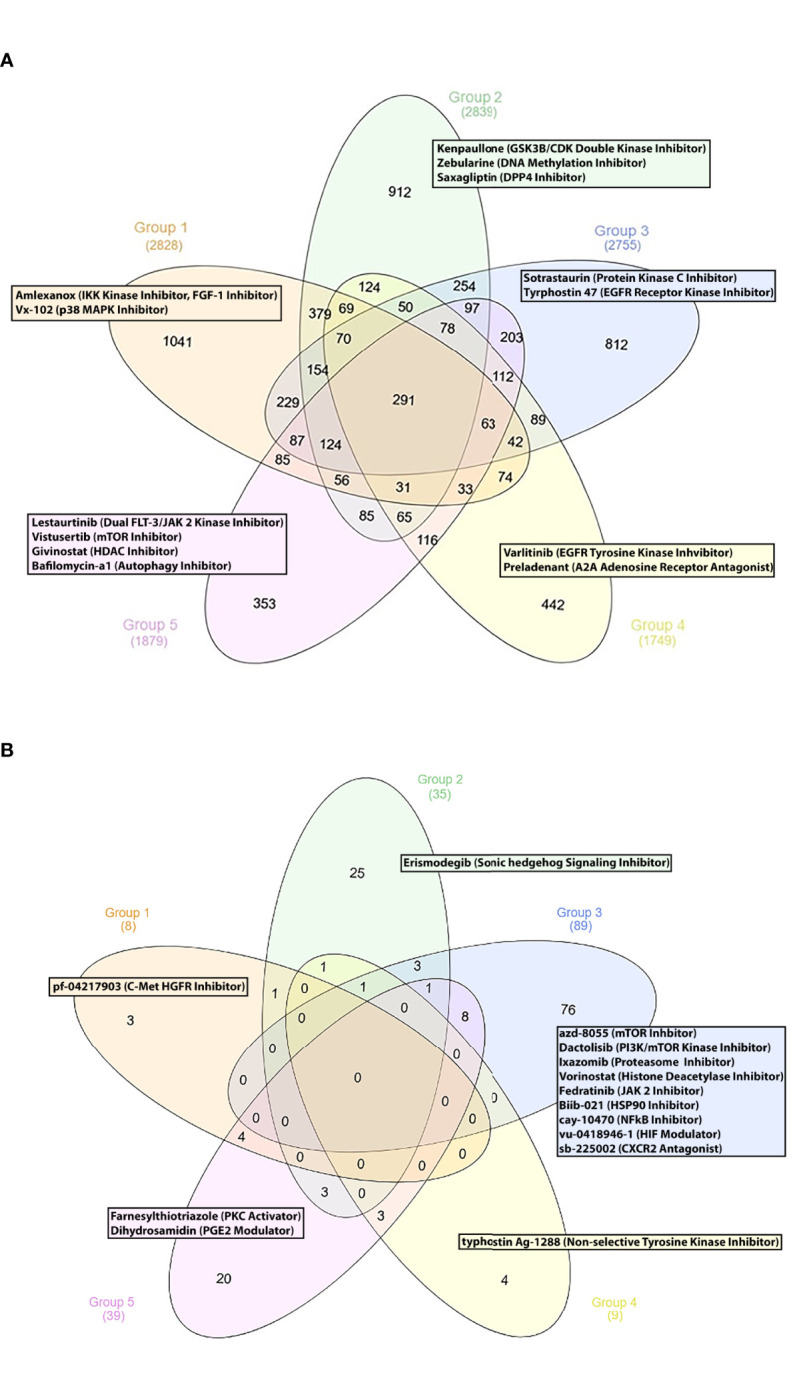
**(A)** Group specific compounds derived from iLINCS platform-based drug repurposing analysis. **(B)** Group specific compounds derived from CLUE platform-based drug repurposing analysis.

Finally, we sought to propose new SLE therapeutic agents. To this end, we used a drug repurposing pipeline identifying patented compounds with potentially unrecognized efficacy in SLE. Using the iLINCS and CLUE platforms, we identified novel compounds that could reverse the previously defined SLE group-specific signatures. To sort out the top perturbagens derived from the iLINCS platform, we applied a negative concordance score cut-off of ≤ -0.5. Regarding the CLUE based analysis, only compounds exhibiting an inhibitory score of ≤ -50 were selected. Lastly, group-specific perturbagens were determined, as shown in the Venn diagram (**Figures 4A, B**). To enhance the performance of our approach, group-specific compounds were ranked, according to their druggability (“druggability prediction”). For this purpose, we used the bioinformatic tool CoDReS (Computational Drug Repositioning Score) (14). Uploading the iLINCS- and CLUE-derived compound lists (which were related exclusively to each SLE endotype) to the CoDReS platform resulted in the re-ranking of the repurposed drugs, according to their biological and pharmaceutical potential (**Tables S6** **–** **S15**).

#### G1 Subgroup

Our analysis indicated the p38 MAP kinase inhibitor vx-102 (17) and the TBK1 and IKK kinase inhibitor amlexanox, as potentially beneficial compounds. Lenalinomide, which has been tested in SLE clinical trials (18), and the c-met-HGFR (hepatocyte growth factor receptor) inhibitor pf-04217903 (19) might also be considered as treatment options for G1 SLE patients.

#### G2 Subgroup

The GSK3B/CDK double kinase inhibitor kenpaullone (20) was found to reverse G2-specific transcriptional patterns. Notably, the antidiabetic DPP4 inhibitor saxagliptin (21), the DNA methylation inhibitor zebularine [used for the treatment of CD4+ T cells mediated uveitis in a murine model (22)], the smoothened receptor antagonist erismodegib [inhibitor of the sonic hedgehog signaling (23)] were also identified as potential therapeutic compounds.

#### Other Subgroups

Concerning G3, we identified numerous potential drug candidates, including the protein kinase c (PKC) inhibitor sotrastaurin (24), the EGFR receptor kinase inhibitor tyrphostin 47 (25), and the mTOR kinase inhibitors azd-8055, wye-125132, ku-0063794, wye-354 and torin-1 (26). Our data also underlined the potential role of the dual PI3K/mTOR kinase inhibitor dactolisib, the proteasome inhibitors ixazomib and mg-132 (27), the histone deacetylase inhibitors (HDACs) panobinostat, vorinostat, dacinostat, apicidin and merck60 (28, 29), and the HSP90 inhibitor biib021 (30) for potential treatment of patients in G3. Based on their pathophysiological relevance, the HIF (hypoxia inducible factor) modulator vu-0418946-1 (31), the NFkB inhibitor cay-10470 (32), the CXCR2 antagonist sb-225002 (33), the JAK2 inhibitor fedratinib (34), might represent promising therapeutic choices for G3 patients.

Moreover, chemical substances, such as the EGFR 2 receptor tyrosine kinase inhibitor varlitinib (25), the A2A adenosine receptor antagonist preladenant (35) and the niacin (vitamin B3) (36) were found to be G4-specific drug candidates.

Finally, small molecules, such as the artemisinin derivative artesunate, a drug applied for malaria (37), the dual FLT-3/JAK 2 kinase inhibitor lestaurtinib (38), the class1/2 HDAC inhibitor givinostat (39), the mTOR kinase inhibitor vistusertib (26) and the autophagy inhibitor bafilomycin-a1 (40) were identified as G5-specific compounds.

## Discussion

Despite advances in our understanding of SLE pathogenesis, selecting the optimal treatment for each individual patient remains a challenge. Herein, we applied a whole blood transcriptome-based molecular taxonomy strategy to stratify SLE patients according to their molecular fingerprints. Leveraging high-throughput computational methods, we exploited patient molecular endotypes to optimize putative therapeutic choices in a personalized approach. Finally, we applied available bioinformatic tools to establish a personalized drug repurposing methodology for the identification of new compounds that could enrich our armamentarium in SLE treatment.

Our data-driven re-stratification approach recapitulated the spectrum of previously identified lupus pathophysiological processes. For example, Banchereau et al. have shown that progression to active lupus nephritis is accompanied by an incremental enrichment of neutrophilic gene expression signatures (41). Accordingly, transcriptional signatures reflective of neutrophil activation defined G4 subgroup in our study, which consisted almost exclusively of active lupus nephritis patients.

Previous studies have highlighted the crucial role of type I IFN signaling in the loss of B cell tolerance and autoantibody production in SLE-prone mice (42). Gene expression signatures indicative of type I IFN production, B cells and plasmablast activation prevail in G5 group, implying the presence of type I IFN-induced autoreactive B cell development.

Incomplete response to existing drugs remains a substantial challenge for SLE patients, while various reasons related both to the disease and to trial design have accounted for the failure of several SLE clinical trials. Exploiting one of the largest drug signature databases to date, iLINCS, allowed us to predict the best patient endotype-specific drug candidates from a pool of currently available therapies and drugs. To this end, Alexander et al. have proposed the proteasome inhibitor bortezomib as a putative therapeutic option for patients with refractory lupus (43). Our unbiased approach indicated that use of bortezomib might be efficacious for the treatment of patients belonging to the “Neutrophil” molecular endotype. Moreover, expression of Syk is increased in SLE T cells and skin lesions of lupus MRL/lpr mice (44, 45), while administration of Syk inhibitors ameliorates kidney injury in lupus-prone mice (44). In this regard, our results suggest that patients in the G3 “Metabolism” subgroup might benefit most from treatment with fostamatinib. Depletion of abnormal plasma cells is considered a potential mechanism of action of the proteasome inhibitor ixazomib (46). In this context, our drug prediction analysis further substantiates the therapeutic relevance of targeting B cell responses in patients’ group G5 (“B-cell” subgroup).

Over the last years, *in silico* drug repositioning studies for SLE have been published, based on gene expression and genetic profiles (47–49). Furthermore, efforts have been made to individualize drug repurposing results, according to the molecular features of lupus patients (49), whereas several studies have applied literature mining approaches, in order to prioritize the most promising compounds (50, 51). Herein, we performed personalized drug repurposing analysis using two robust, high-throughput platforms (iLINCS and CLUE). Notably, the top-ranked compounds were assessed not only through extensive literature review, but also according to their “druggability” profile. Activation of PI3K/Akt/mTORC1 signaling pathway characterizes T cells of SLE patients (52). In addition, pharmacological dampening of PI3K signaling in lupus-prone mice provides evidence for the therapeutic potential of targeting PI3K/Akt/mTORC1 pathway in SLE (52). Similarly, our findings indicate that several inhibitors of the PI3K/mTOR pathway (azd-8055, dactolisib) might be promising therapeutic options for patients belonging to the “Metabolism” (G3) group. Aberrant type I IFN and IFN-γ signaling and the encouraging results from baricitinib phase 2 study in SLE provide a clear rationale for targeting the JAK/STAT pathway in SLE (53). To this end, administration of the JAK2 inhibitor fedratinib, identified by our approach as an appropriate treatment for patients in G3 group, might also confer therapeutic benefit.

Certain limitations of our study deserve acknowledgment. First, the vast majority of patients included in this study were receiving immunosuppressive treatment at sampling, thus therapy-induced immunosuppression may be mirrored in the whole blood transcriptional profile, altering the expression of essential pathophysiological mechanisms. Moreover, our *in silico* drug prediction strategy is an explorative approach and additional *in vitro* and *in vivo* studies are clearly required to confirm our findings. Results of the phase III clinical trials BLISS-LN (54) and AURORA 1 (55) have shown a clinical benefit of adding belimumab or voclosporin, respectively, on top of standard-of-care in patients with lupus nephritis. Regarding the molecular complexity of the disease, also underscored by our findings, these studies might denote the need towards combination treatment approaches. Obviously, further drug combination prediction analysis might be useful to explore new avenues for SLE treatment.

In summary, we present a molecular taxonomy-based pipeline to guide therapy and identify new compounds for patients with SLE, based on a comprehensive, in-depth analysis of the transcriptome. These data need to be further validated and tested in preclinical models of SLE and in longitudinal clinical studies.

## Data Availability Statement

Publicly available datasets were analyzed in this study. This data can be found here: https://ega-archive.org/studies/EGAS00001003662.

## Author Contributions

PG, DN, and SD performed the analyses. PG, SD, and DN drafted the manuscript, with contribution of all authors. GS and Anastasia Filia contributed in figure generation. Antonis Fanouriakis, GB, and EF evaluated clinical data and participated in the analyses and interpretation of the data. DB, GB, and Antonis Fanouriakis conceived, critically revised and oversaw the study and the writing. All authors contributed to the article and approved the submitted version.

## Funding

This work was supported by grants from EU (SYSCID grant agreement number 733100), ERC (LUPUSCARE grant agreement number 742390) and the Foundation for Research in Rheumatology (FOREUM)all to DB.

## Conflict of Interest

The authors declare that the research was conducted in the absence of any commercial or financial relationships that could be construed as a potential conflict of interest.

## Publisher’s Note

All claims expressed in this article are solely those of the authors and do not necessarily represent those of their affiliated organizations, or those of the publisher, the editors and the reviewers. Any product that may be evaluated in this article, or claim that may be made by its manufacturer, is not guaranteed or endorsed by the publisher.

## Supplementary Material

Supplementary Figure 1 Modules (pink to indian-red) of commonly regulated transcripts as identified by CoCena2 analysis and heatmap depicting the group fold changes (GFC) of each sample per module. The identified transcript modules were illustrated in the annotation color bar on the right side of the heatmap. The patients analyzed in our study were shown in the x-axis of the heatmap. GFC were defined for each gene by computing the mean expression of a gene across all samples, followed by calculating the sample specific fold change of the gene expression from the overall mean. Then, the GFC of all genes within each module were added and divided by the total number of genes of each module, returning the GFCs of each sample per module. Briefly, the color intensity represented the relative magnitude of the expression of each gene module per SLE patient. GFC denotes group fold change; GB followed by number denoted the patient identifier according to our anonymous coding system.

Supplementary Figure 2 Barplots demonstrating the distribution of demographic features as well as the frequency of NPSLE history, Antiphospholipid Syndrome (APS) history, serum anti-DNA antibodies positivity, antiphospholipid antibodies positivity across the patients groups. *:p<0.05; **:p<0.01 in Kruskal-Wallis test, Chi-squared test.

Supplementary Figure 3 Barplots displaying the treatments the patients were receiving at the sampling timepoint. Cyclophosphamide and MMF were the most commonly used treatments in the G4. *:p<0.05; **:p<0.01 in Kruskal-Wallis test, Chi-squared test.

Supplementary Figure 4 Clusters of drugs signatures (Cluster 1-4) as identified by k-means clustering according to the ΔNES scores. ΔNES score was defined as the difference between the NES from the downregulated gene set and the NES from the upregulated gene set for each drug signature. Utilizing the calculated ΔNES scores, drug signatures were next grouped using the k-means clustering method into 4 clusters, which were shown on the right side of the heatmap. The heatmap visualized how each of the 4 identified drug clusters were enriched in the specific patient groups. A group specific predominant enrichment of a drug cluster indicated that the drugs included in the drug cluster of interest might be the most potent drug candidates for the specific patient group. Briefly, cluster 4 contained drug signatures that were predicted to most efficiently reverse the transcriptional aberrations of G4. Accordingly, drug cluster 3 might contain the best drug candidates for group G5, whereas drug cluster 1 included drug signatures that might most effectively counteract the G3-specific transcriptional changes.

Supplementary Figure 5 Elbow method identified optimal number of drug clusters for k-means clustering.

Supplementary Table 1 Drugs that are currently used in the treatment of SLE or evaluated in SLE clinical trials and their gene expression signatures are included in iLINCS sublibraries.

Supplementary Table 2 Gene expression signatures of the drugs of table 1. that are listed in the iLINCS prediction databases.

Supplementary Table 3 Ranking of specific drug related signatures for each SLE patients’ molecular endotype, according to ΔNES score.

Supplementary Table 4 Drug related signatures with ΔNES scores for patient group G4A.

Supplementary Table 5 Drug related signatures with ΔNES scores for patient group G4B.

Supplementary Table 6 Ranking of the compounds derived from the iLINCS platform through the CoDReS platform. This analysis was performed for SLE patients’ molecular endotypes separately.

Supplementary Table 7 Ranking of the compounds derived from the iLINCS platform through the CoDReS platform. This analysis was performed for SLE patients’ molecular endotypes separately.

Supplementary Table 8 Ranking of the compounds derived from the iLINCS platform through the CoDReS platform. This analysis was performed for SLE patients’ molecular endotypes separately.

Supplementary Table 9 Ranking of the compounds derived from the iLINCS platform through the CoDReS platform. This analysis was performed for SLE patients’ molecular endotypes separately.

Supplementary Table 10 Ranking of the compounds derived from the iLINCS platform through the CoDReS platform. This analysis was performed for SLE patients’ molecular endotypes separately.

Supplementary Table 11 Ranking of the compounds derived from the CLUE platform, through the CoDReS platform. This analysis was performed for SLE patients’ molecular endotypes separately.

Supplementary Table 12 Ranking of the compounds derived from the CLUE platform, through the CoDReS platform. This analysis was performed for SLE patients’ molecular endotypes separately.

Supplementary Table 13 Ranking of the compounds derived from the CLUE platform, through the CoDReS platform. This analysis was performed for SLE patients’ molecular endotypes separately.

Supplementary Table 14 Ranking of the compounds derived from the CLUE platform, through the CoDReS platform. This analysis was performed for SLE patients’ molecular endotypes separately.

Supplementary Table 15 Ranking of the compounds derived from the CLUE platform, through the CoDReS platform. This analysis was performed for SLE patients’ molecular endotypes separately.
