# Supplementary figures and images for: Molecular Taxonomy of Systemic Lupus Erythematosus Through Data-Driven Patient Stratification: Molecular Endotypes and Cluster-Tailored Drugs
Source: Front Immunol. 2022 May 9;13:860726. doi: 10.3389/fimmu.2022.860726 (PMC9125979; doi:10.3389/fimmu.2022.860726)

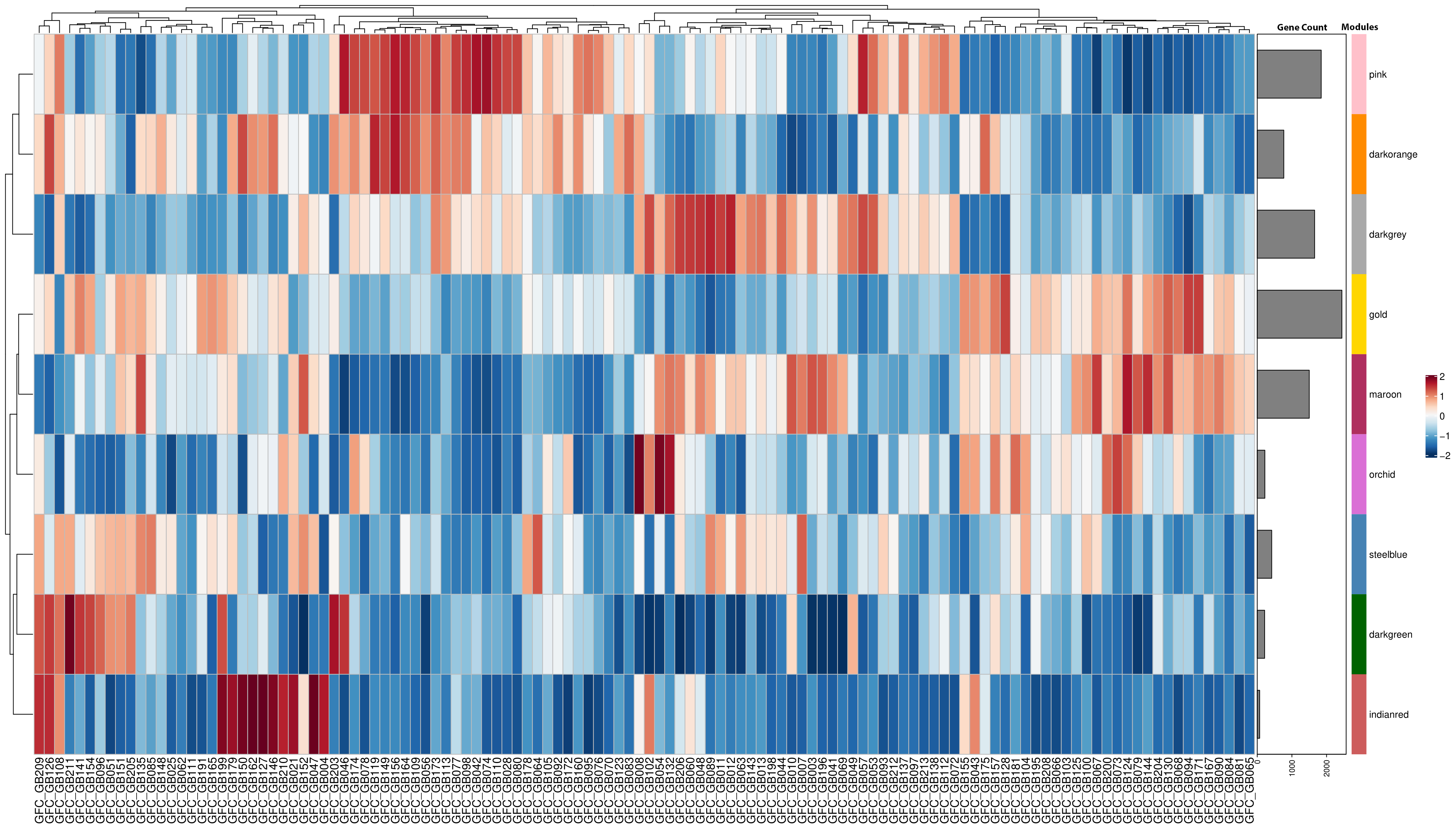

Supplement: Supplementary Figure 1 — Modules (pink to indian-red) of commonly regulated transcripts as identified by CoCena2 analysis and heatmap depicting the group fold changes (GFC) of each sample per module. The identified transcript modules were illustrated in the annotation color bar on the right side of the heatmap. The patients analyzed in our study were shown in the x-axis of the heatmap. GFC were defined for each gene by computing the mean expression of a gene across all samples, followed by calculating the sample specific fold change of the gene expression from the overall mean. Then, the GFC of all genes within each module were added and divided by the total number of genes of each module, returning the GFCs of each sample per module. Briefly, the color intensity represented the relative magnitude of the expression of each gene module per SLE patient. GFC denotes group fold change; GB followed by number denoted the patient identifier according to our anonymous coding system. [file Image_1.tiff]

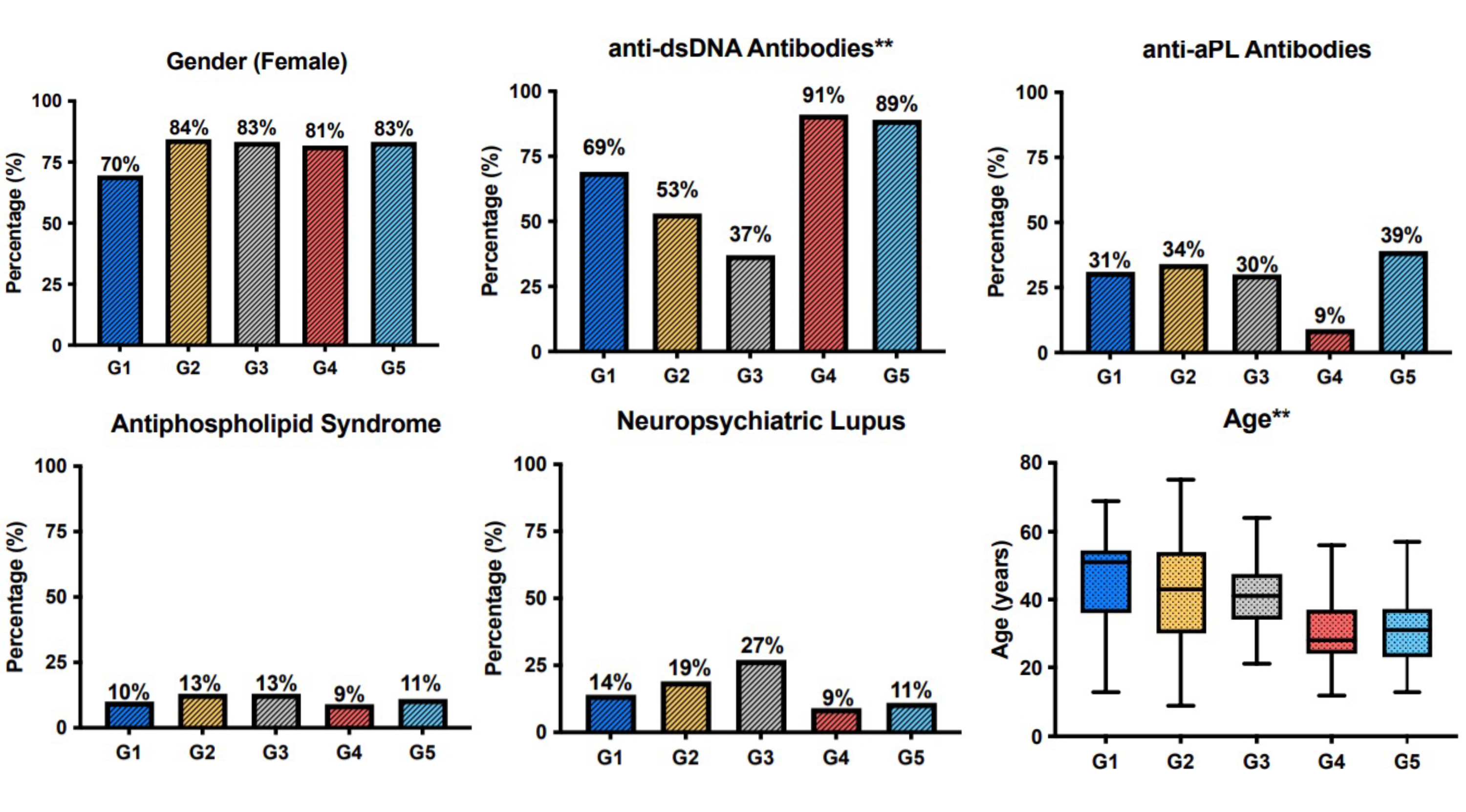

Supplement: Supplementary Figure 2 — Barplots demonstrating the distribution of demographic features as well as the frequency of NPSLE history, Antiphospholipid Syndrome (APS) history, serum anti-DNA antibodies positivity, antiphospholipid antibodies positivity across the patients groups. *:p<0.05; **:p<0.01 in Kruskal-Wallis test, Chi-squared test. [file Image_2.tif]

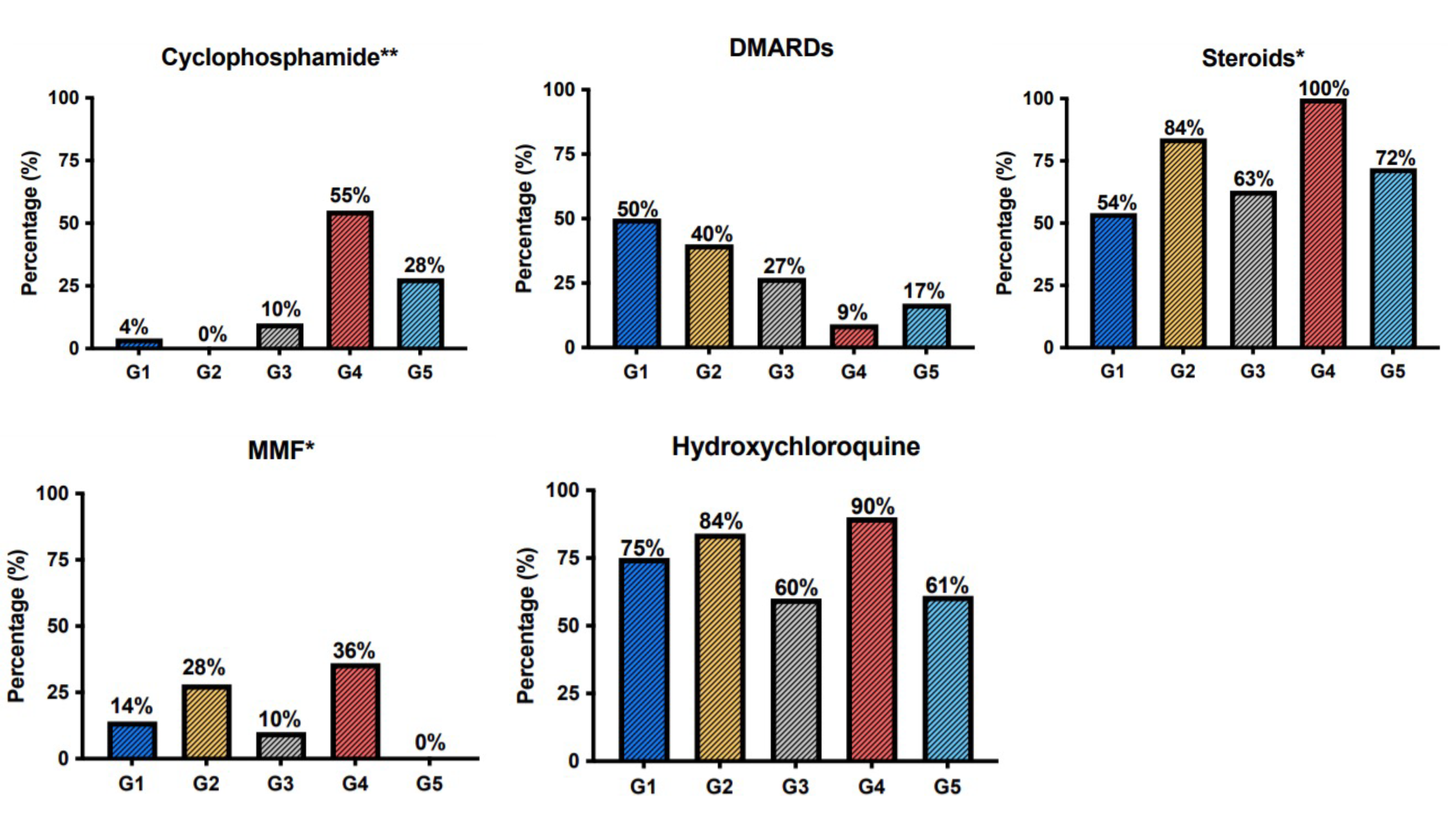

Supplement: Supplementary Figure 3 — Barplots displaying the treatments the patients were receiving at the sampling timepoint. Cyclophosphamide and MMF were the most commonly used treatments in the G4. *:p<0.05; **:p<0.01 in Kruskal-Wallis test, Chi-squared test. [file Image_3.tif]

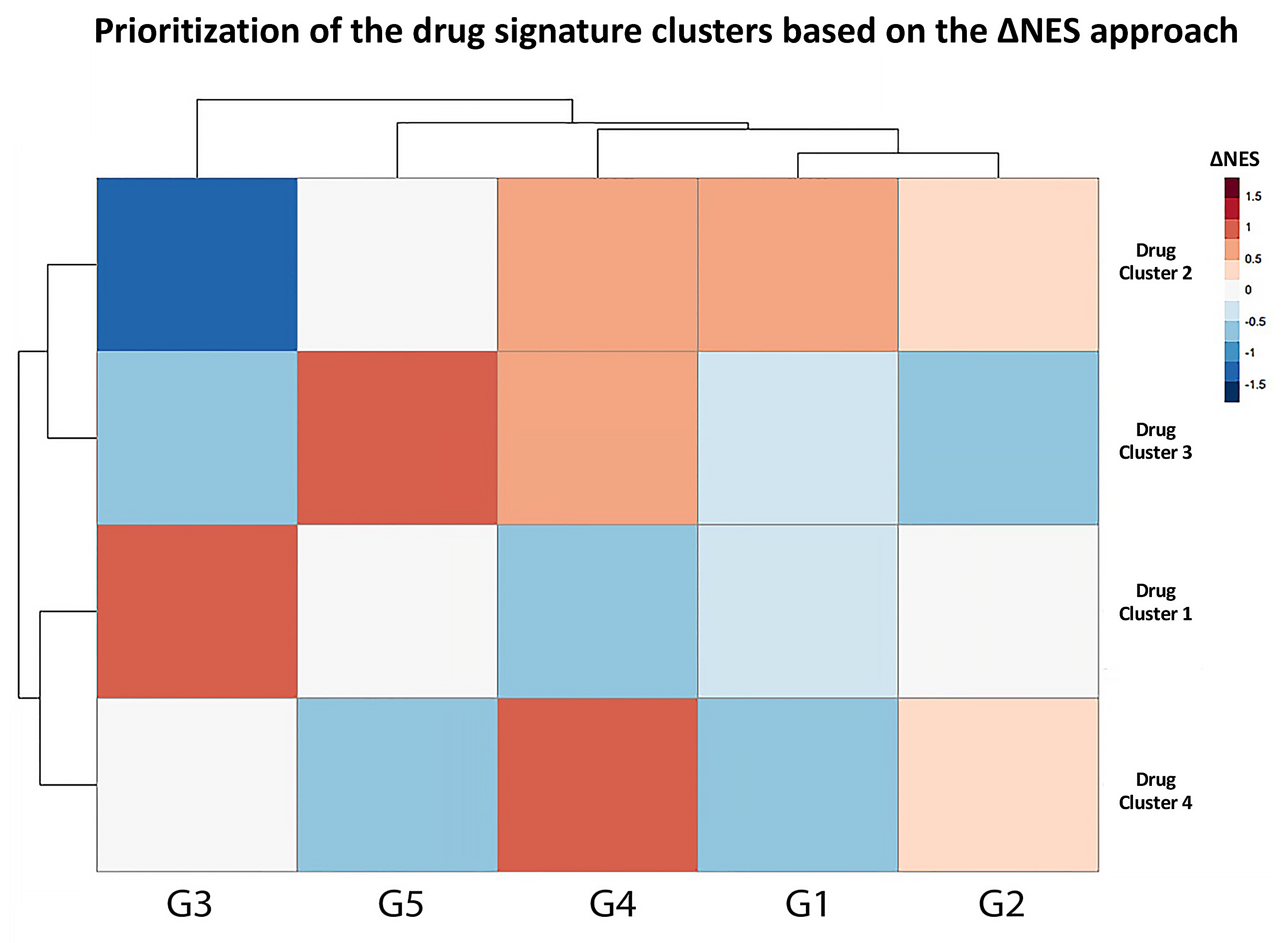

Supplement: Supplementary Figure 4 — Clusters of drugs signatures (Cluster 1-4) as identified by k-means clustering according to the ΔNES scores. ΔNES score was defined as the difference between the NES from the downregulated gene set and the NES from the upregulated gene set for each drug signature. Utilizing the calculated ΔNES scores, drug signatures were next grouped using the k-means clustering method into 4 clusters, which were shown on the right side of the heatmap. The heatmap visualized how each of the 4 identified drug clusters were enriched in the specific patient groups. A group specific predominant enrichment of a drug cluster indicated that the drugs included in the drug cluster of interest might be the most potent drug candidates for the specific patient group. Briefly, cluster 4 contained drug signatures that were predicted to most efficiently reverse the transcriptional aberrations of G4. Accordingly, drug cluster 3 might contain the best drug candidates for group G5, whereas drug cluster 1 included drug signatures that might most effectively counteract the G3-specific transcriptional changes. [file Image_4.tif]

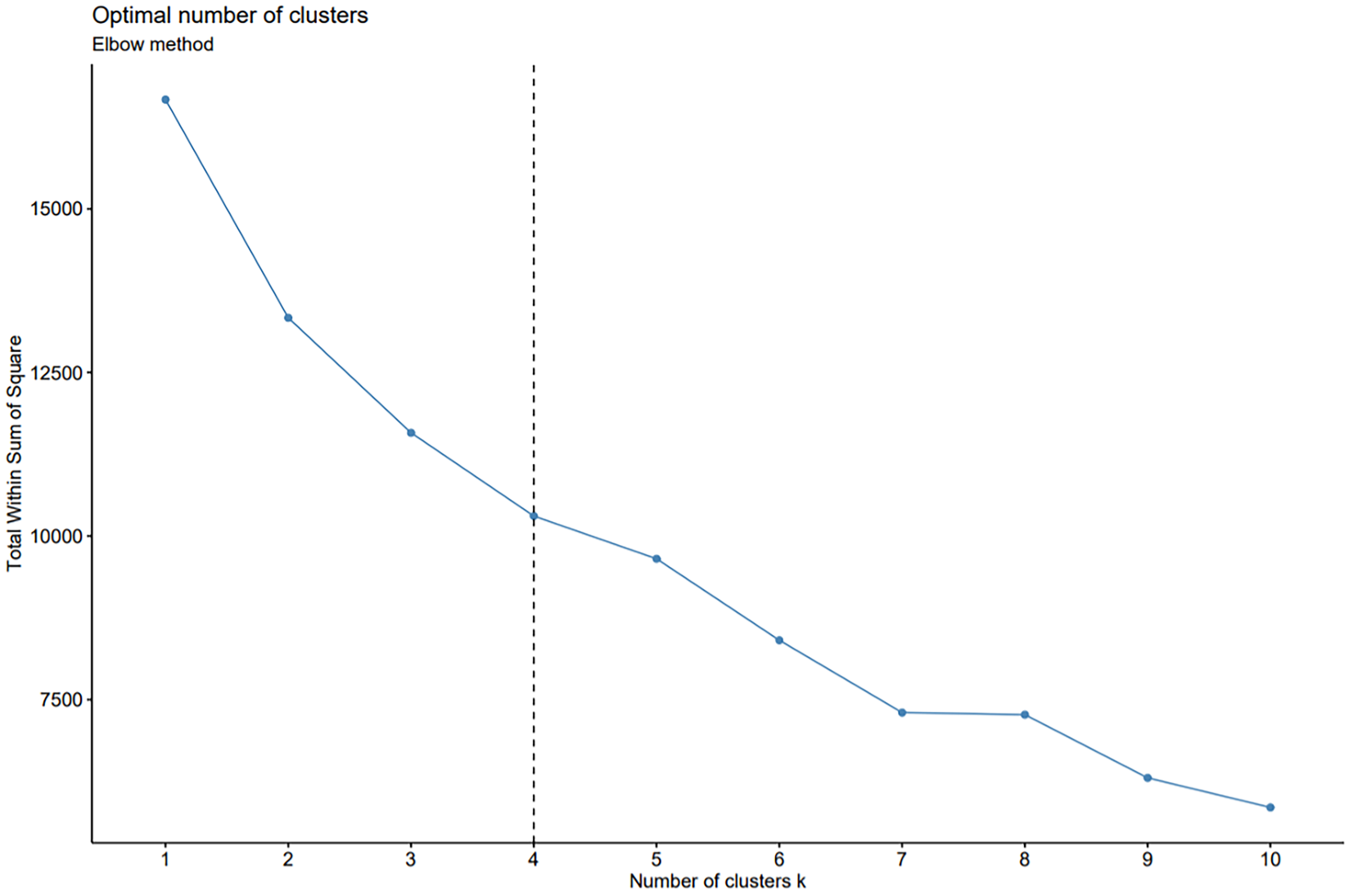

Supplement: Supplementary Figure 5 — Elbow method identified optimal number of drug clusters for k-means clustering. [file Image_5.tiff]
